# From transcendental egology to orientation theory: Toward a mereological foundation for the different senses of the “self” in conscious experience

**DOI:** 10.3389/fpsyg.2022.1069448

**Published:** 2022-12-02

**Authors:** Joan González Guardiola

**Affiliations:** Department of Philosophy and Social Work, University of the Balearic Islands (UIB), Palma, Spain

**Keywords:** conscience, self, orientation theory, Edmund Husserl, phenomenology

## Abstract

In the present work, we aim to make a contribution to the origins of the notion of “minimum self” in Husserl's phenomenology. Starting from the difference between the philosophy of the subject and the philosophy of the self, the aim of this research is to show that the Cartesian association between both philosophies would not exactly correspond to the conception of the self, as we find it in Edmund Husserl's works. With this, we intend to nuance Heidegger's accusation of Husserl's “Cartesianism,” At the same time, we show how a detailed analysis of the “senses of the self” in Husserl's phenomenology allows extracting the notion of “minimal self” as it has been introduced in the current and lively debate between psychiatry and phenomenology. In our research, we also show that in order to move the theory of the transcendental ego toward the theory of the orientation of the life of consciousness, it is necessary to consider the foundation of the concepts of ego in the technical vocabulary of the formal mereology of the Husserl's third “Logical Investigation.”

## Introduction

Many modern approaches to subjectivity, especially since Descartes's theory, share the perception that subject and ego are intimately linked, as if they were the same problem formulated in two different ways. Nonetheless, the history of metaphysics has shown that it would be wise not to simply accept this assumption, which can lead to automatically associate reflections on the ego and the subject: first, both are differently articulated within the framework of the structure of predication^1^. The process that produces a progressive substantivization of the “I” pronoun in the history of metaphysics, with it being absorbed in the enunciative proposition as what lies beneath all enunciation, has been described (to a greater or lesser degree of success) by M. Heidegger^2^. However, even Heidegger warns that in principle, nothing of “I-hood” (*Ichheit*) would necessarily be found in the formal apophantic category of the *subiectum*. The *subiectum*, a Latin translation of the Greek term ύπ*oκε*íμ*ενo*ν in Aristotle's logical treatises, fails to retain any trace of “I-hood”; it simply and formally indicates the position in the enunciation of that which is predicated. In Aristotle's treatises, tracing back from the “what” is predicated to the “of that which” is predicated does not culminate in the ψ*υχ*ή (and even less so in the ν*o*υς), but rather in the category of the *o*ύσíα^3^. The area of problems with the *o*ύσíα is not particularly concerned with the specific case of the “living thing” (ζωή), the treatment of which is confined, for Aristotle, to interrogating the soul (Π*ερ*í ψ*υχ*ής). It would seem that no fundamental crossover occurs in Aristotle's treatises between the theory of being (being as being) and the specific case of the theory of “living things,” to which specific investigations into the soul are dedicated. Indeed, it appears that for Aristotle, the soul (ψ*υχ*ή) being the *o*ύσíα of life simply means that everything predicated about a living thing ultimately finds its logical “subject” in the soul^4^. The implacable nature of the πρώτη *o*υσíα with regard to predication is linked, according to Aristotle, and not so much with ψ*υχ*ή, but rather with the ineffable nature of the individual (whether this individual has life or not), since the “something of something” (τí κ*ατ*ά τ*ινo*ς) structure inherent to all demonstrations collapses in the predication of individuals. Heidegger would use this ineffable and impredicative nature of individuals, as per Aristotle, to link the concept of πρώτη *o*υσíα to the “existence” of being and to the idea of life as facticity (*Dasein*), more than with any idea relating to understanding life as “self-consciousness^5^.” Descartes proposed linking the soul to the idea of self-consciousness, by discerning an apodeictic relationship between ego and *cogitatio*. Through the Cartesian establishment of the ego as “I think,” and the position of this “I think” as a substance (*res cogitans*), it is possible to constitute it as “*subiectum*” of all possible predication. The fusion process between the I and the subject could be defined in three steps: (1) I is always “I think,^6^” (2) “I think” is interpreted as “*substantia*,^7^” and (3) the thinking substance is the “subject” of all possible predication. Not only this, as the thinking substance is the condition of possibility of the coherent unit of representation for all predicates, the “I” becomes a guarantee for the coherence of the synthesis processes of sensitive multiplicities in their respective conceptual units^8^.

Heidegger also includes the concept of consciousness from his professor, E. Husserl, in this hermeneutic reconstruction of the process by which the “I” and “subject” converge in the history of metaphysics. In Heidegger's assessment, while setting out certain fundamental differences between Husserl and Descartes, “Husserl completely moves in the direction of Descartes”^9^. In the winter term of 1923/24 and the summer term of 1925, Heidegger gave critical presentations on the phenomenology of his professor, where he declared excessive tethering to Descartes' premises and intentions in Husserl's initial phenomenology project. Heidegger proposed moving away from these premises and intentions through “radicalizing” the phenomenological method, in a shift toward ontology^10^. After the publication of “Being and Time” in 1927, for both Heidegger and Husserl, the name “Descartes” became the symbol of a barely disguised confrontation between two ideations of phenomenology (viz., transcendental phenomenology and hermeneutic phenomenology), whose paths would remain separate to the present day.

Nonetheless, the purpose of this article is not to review Heidegger's criticism of Husserl's concept of phenomenology from his lectures in the 1920s^11^, but rather to directly show how Husserl's phenomenological approach to the notion of the “I” contains many nuances, so much so that it enables us to go beyond the strict limitation of the “I” to the topic of subjectivity. In this way, it will also attempt to show how the many nuances in Husserl's notion of the “I” provide ways to describe the many experiences of altered consciousness, with the basis of the debate on the existence of the “minimal self” offering an explanation for many phenomena of interest to psychiatry and neuroscience^12^.

## The three senses of “ego” in “Cartesian Meditations”

Much of Heidegger's criticism is based on the idea that at no point does Husserl determine the “conscious being.” However, Husserl does indeed determine the “conscious being” from the start, through Brentano's concept of “intentionality”: the conscious being is characterized by intentionality; “being” conscious means being composed of intentional experiences. For Heidegger, this does not suffice to characterize the conscious being since according to his interpretation, Husserl's concept of intentionality remains directly focused on “theoretical behavior” (*theoretisches Sichverhalten*)^13^: intentionality would be limited to providing representations when judging, wanting, loving, etc. This is a highly limited concept of intentionality based in large part on Husserl's presentation of it in “Ideas I,” which does not include the presentation and explanation in later works, such as “Cartesian Meditations.”

In sections [31]–[33] in “Cartesian Meditations,” Husserl meditates on the meanings of the “I” in the field of transcendental egology^14^. In section [30], he sets the mereological inseparability of the ego and processes constituting life. This mereological inseparability is always fundamental to Husserl's phenomenology: on the basis of this mereological inseparability of the ego and life experiences, intentionality must always be interpreted as correlation^15^. Section [30] thus provides the immediate context for interpreting the meanings of the ego in the subsequent three sections: intentionality is understood as the vehicle that traverses the correlation between the object of experience and its different manners of givenness. In this sense, the transcendental ego is inseparable from the processes making up life: both (ego and experiences) lie solely in the ambit of correlation. It should be noted that when Husserl uses the word “separable/inseparable,” one needs to draw on the meaning of these concepts from his sole operational use of the expression “ontology” which, far from being a mystery or grandiloquence, is always posited as formal mereology^16^. Therefore, intentionality defines and delineates the “how” of *a priori* correlation, although it loses all interest in phenomenological research outside of this. More than an essential note of “being conscious” of consciousness, intentionality gains its fundamental importance for phenomenological research as it defines the “how” of correlation^17^. Thus, on the basis of this understanding of intentionality, Husserl defines the first of the phenomenological meanings of the ego: the I-pole (*Ich-Pol*).

It would be a mistake to take the polarity that has accompanied Husserl's analyses of intentionality from the beginning as implicit. Indeed, there is no language of polarity in “Logical Investigations,” and its highly precarious appearance in “Ideas I” has a specific and fairly circumstantial meaning^18^. The language of polarity to describe the “how” of correlation (and, therefore, to define the specificity to which we refer when we say that correlation is “intentional”) is introduced in the descriptions of § [25] in “Ideas II.^19^” The first uses of the language of polarity arise from certain ambiguities that Husserl comes across when attempting to describe the directionality of intentionality (especially in the case of the phenomena of attention), such as a ray (*Strahl*)^20^. In this way, the ray comes before polarity, which serves as a descriptive support or complement. These ambiguities come from the fact that in effect, the ego is both the ray and the point of origin (*Ausgangspunkt*) of the ray. By interpreting the ray of attention as a structure of polarity, certain advantages are won when dealing with the difficult descriptions of intentional life: (1) First, a better distinction is made possible between “I” and “consciousness,” and the understanding of the former as a part of the latter is enabled (in the language of mereology in LU III, we would say a “non-independent part”). The idea of “pure consciousness” uses ego as the “whole” of the intentional ray, while the idea of the “pure ego” (for descriptive needs, the first meaning as “I-pole” will be introduced)^21^ uses ego as merely a “part” of the former, more specifically to the part that represents the non-separable, identical, and featureless center on which the variations and modifications of all intentional correlation hinge^22^. (2) Second, there is an immediate presumption of the necessary implacable *heterogeneity* between the extremes of correlation (the poles)^23^. (3) Third, *double directionality* between correlation poles is better described^24^. (4) Last but not least, the introduction of the language of polarity (which Husserl derives from physics of the era) paradoxically enables the recovery of etymological specificity preserved in the choice of the term “intentionality.” Indeed, *intentio* is derived from the Latin word *tendere*, which we could translate as both “stretch” and “aim/direct.” Both translations are possible, thanks to the Latin verb *tendere* having two participles: *tensus*, from where it gets its relationship to the field of forces (in today's physics, we would say “elastic forces”), and the other from the convergence with the Latin verb *temptare* (probe or touch, but also strike or prey), which gives the participle *tentus*, whose frequentative form is tentare^25^, from where we could get its relationship to the field of attention and, from this, to the entire semantics of “aim/direct.^26^” Thus, we see the double play in the notion of *intentio*: the play between “tighten/extend.” The grouping of both semantic fields into one makes perfect sense yet should not mean we overlook the relative separateness or isolation of both. In a representation of vector for any elastic forces, we obtain a tension for which the notion of “direct” would only be metaphorically applicable, for example when we attribute a “tendency” to the balance of bodies after the forces of tension cease to act on it. The “direct” force of intentionality as a mental phenomenon has nothing (or, at least initially, little) to do with the “tension” to which an elastic body is subjected when we place a force from one or both of its poles. In turn, the phenomena of attention may be perfectly described based on their self-contained properties, turning their back on an explanation in mechanical terms (here, physiological), without losing any of their meaning: in visual perception, the physiological process whereby the lens flattens or enlarges based on the size of the perceived object, thus enabling one of the key fundamental elements in depth perception^27^, may be described without reference to the fact that the I-pole, in its constituent mobility, attentively encircles this object, instead of the other; the “direct” element mobility for phenomena of attention seems to be explained relatively independently from the logic of “tensions” between physical forces, and its description is always sustainable outside them. Nevertheless, the relative isolatable nature of the field of “stretch–tighten” with regard to “direct–expand,” both inherent to and contained in the notion of *intentio*, should not lead us to overlook the possibilities that both elements occur in combination in the field of certain specific experiences of “directions that tighten” forces, on the one hand, and “tensions that accommodate” a direction, on the other hand; for example, in a description of archery, both directions occur in combination and overlap in an authentic layering: the experience of direction in archery simultaneously contains an overlap between a set of forces and a postural heterogeneity, including *on* a phenomenon of attention^28^.

These four advantages (mereological elucidation of the distinction/association between ego and consciousness; the establishment of the irreducible heterogeneity of the correlation poles; the acceptance of double directionality as recognition of the active–passive dimension of correlation; and for the phenomenological notion of intentionality, the recovery of its dynamic dimension as *life*) lead directly to the second sense of the ego: the ego as a “substrate of habituality” (*Substrate von Habitualitäten*)^29^. This second sense depends on the fact that the I-pole is not separable (as a non-independent part) from the content of consciousness that it directs. Thus, the permanence of the I-pole pulls along the diversity of acts that hinge on it. Nonetheless, these acts cannot occur without organization (here, centralization) into a permanent pole to which they adhere. The pole gains continuity through the diversity of acts, and acts settle on the permanence of the identical pole, becoming a substratum (through the ever-active consciousness of time). The I-pole and I-substrate thus seem to be abstract parts (non-independent) of a whole for Husserl^30^. In this regard, two questions arise: (1) What type of dependency relationship do the I-pole and I-substrate establish? (2) Which “whole” do both senses of the ego form part of?

The second question is the truly relevant one. Husserl answers the first question without hesitation through a bilateral existential dependency model: A and B are existential and bilaterally dependent if and only if it is logically impossible for A to exist without the existence of B, and it is logically impossible for B to exist without the existence of A^31^. In other words, any pole is such from intentional lived experience, and the concept of “intentional lived experience” always involves an ego polarity. However, in the present study, this ontological (mereological) relationship is simply thought of as referring to the abstract “I-pole” and “I-substrate” genders. If both parts are simply thought of as abstract (non-independent), we do not seem to gain much terrain for the effective description of intentional lived experiences and their relationship to specific reality. By referencing “substrate” (*Substrat*) and “sedimentation” (*Niederschlag*), Husserl is not merely thinking of abstract objects and conceptual relationships, but rather of structures that constantly overlay in the courses of time for specific lived experiences. This can be clearly seen when Husserl allows the introduction of affections (*Affektion*) between the sedimentations of the I-pole: the I-pole contains a passive habituality for bodily affections^32^. This makes the I-pole and I-substrate pairing a directly linked pair to specific courses of time and concrete bodily passivity. Indeed, Husserl perceives the I-pole through the following original analogy (*ursprüngliche Analogie*), which would become fundamental to Husserl's theory of the ego: the I-pole is to act (intentional lived experiences) in a way the lived body (*Leib*) is to sense phenomena: a center^33^. In the same vein, as any bodily affection immediately obtains its location with regard to the lived body, not being able to occur separately from the orientation with regard to the latter (of which it is part), intentional lived experiences in all their diversity have an abstract I-pole at their center^34^. Nonetheless, with the introduction of bodily affections in intentional life, centralized both in the lived body and the I-pole, we now move into the terrain of the truly relevant second question regarding the whole, where the pole and the substrate form abstract parts: the monadic ego and the third sense of the self. Husserl chooses Leibniz's concept of the monad to answer the question of “full concretion” (*volle Konkretion*) of the ego as everything of which the pole and substrate would only be abstract parts. The monadic ego responds to the question of possessive pronominalization from the sphere of concrete consciousness: what contents of consciousness can I accurately say are mine? For Husserl, mine, what belongs to me (*my* hand, *my* pain, *my* imagination, *my* perception, *my* desire, etc.) constitutes a sphere (*Sphäre*) or a field (*Feld*) to which only I can oppose the idea of “not mine” as that which does not belong to me. Thus, a difference and articulation are established between the perimeter sphericity of the I-monad in its full concretion and the horizontality of the “intentional ray.” In contrast to the I-pole, the monad outlines an area, field, or sphericity whose perimeter interiority is also permanently mutable and remains unified by the idea of “property” (*Eigenheit*): the comprising content is *my* content; this precisely characterizes the idea of the monadic ego^35^. Only this I-monad, the only concrete one, vaguely concurs with the idea of possessive pronominalization.

## Husserl's three senses of the ego and the concept of the “minimal self”

It is largely as a response to certain recent theoretical proposals, all with a highly diverse nature^36^ which posit the elimination of the ego given its illusory nature, that Dan Zahavi has suggested the notion of the *minimal self* since the first decade of the 21st century. The term has proved highly successful in the field of psychiatry, and this success comes not so much from a defense against “eliminativist” attempts, but rather from the interdisciplinary work between phenomenology and psychiatry that Zahavi has spent years cultivating and consolidating. As a result of this ongoing work (which is not free from controversies), certain researchers have reached the conclusion that acceptance of a “minimal self” enables us to better explain the lived experiences described by certain patients with psychotic and schizophrenic episodes^37^. The idea of the “minimal self” refers to a property of experience flow: the intentional lived experiences already contain a “for-whom” perspective (in Husserlian terms, this is called the I-pole as the inseparable center of lived experiences). This “for-whom” belongs to the lived experience itself within its structure and does not arise from reflexive acts that revert to and project it. Reflexive consciousness does not turn on itself, in an*a posteriori* attributive description, for example, “When I perceive this house, I am what I perceive,” but rather in “seeing the house” a “for-whom” occurs from seeing it. If it is appropriate to include the “substrate of habituality” in the perception of the house (whereby it is not the first house I am seeing, I know it has windows and doors and it matches the other events of “seeing houses” that have occurred in my life), it is also appropriate that the givenness of the house is such “for me” that I am in that position, walking around it, and it offers me ever newer foreshortening. All these foreshortenings are centralized in a perspective on which they hinge, in the form of the I-pole^38^. This pre-reflexive ego, for which the house exists even before I am asked for whom it exists (i.e., prior to the ego, whether the house is part of the sphere of my property), may be perfectly identified with the first and second egos in their mereological inseparability, as Husserl posits in §§ [31]–[33] in “Cartesian Meditations.” In turn, one should also explore the possibility that it is precisely in dislocations of mereological relations between the first two senses of the ego, and the whole of which they are part, where one could satisfactorily explain the first-person descriptions of individuals who state they have psychotic or schizophrenic experiences^39^. In large part, these lived experiences are described as experiences containing relevant components of *disorientation*, specifically with regard to the interaction between the three senses of the ego set out earlier. Thus, it is possible to interpret depersonalization in terms of disarticulation between the I-pole and the substrate of habituality, which Husserl himself deems the basis of the personal ego.

Nonetheless, the key to interpreting the ego not as the *producer* of intentional life orientation, but rather as its *outcome* (an outcome that would fail in the case of lived experiences arising from the need of the “minimal self,” in terms of intelligibility in the explanation of anomalous lived experiences), can be found in the analogy of the two centralizations of intentional life and bodily life. According to Husserl, both centralizations (intentional life in the I-pole and bodily life in the lived body) are generated based on the orientation (*Orientierung*), as something already present in any originating givenness. It would thus not be the ego that orients, but rather the ego would be the result of the orientation process for intentional life, and, by analogy, it would not be the lived body that would be oriented, but rather the lived body would be the result of the orientation of bodily life^40^. This displaces the senses of the ego from transcendental egology to the possibility of a phenomenological theory of life orientation which, in large part, would remain open to development^41^.

## Data availability statement

The original contributions presented in the study are included in the article/supplementary material, further inquiries can be directed to the corresponding author.

## Author contributions

The author confirms being the sole contributor of this work and has approved it for publication.

## Funding

This article is part of the work developed in the Projects- Fenomenología del cuerpo y experiencias del gozo (Ministerio de Ciencia e Innovación, Proyectos de Generación de Conocimiento 2021, PID2021-123252NB) and Inner Speech in Action: New Perspectives (Ministerio de Ciencia e Innovación/Agencia Estatal de Investigación, PID2020-115052GA-I00).

## Conflict of interest

The author declares that the research was conducted in the absence of any commercial or financial relationships that could be construed as a potential conflict of interest.

## Publisher's note

All claims expressed in this article are solely those of the authors and do not necessarily represent those of their affiliated organizations, or those of the publisher, the editors and the reviewers. Any product that may be evaluated in this article, or claim that may be made by its manufacturer, is not guaranteed or endorsed by the publisher.

## Footnotes

^1^In the structure of predication, “subject” (*subiectum*) is a noun while “I” is a pronoun. The fact that in terms of grammar, pronouns are a subcategory of nouns, which does not evade the need to clearly differentiate between the nouns as subjects of predication and the specific functions granted to pronouns, especially if they are personal pronouns.

^2^Heidegger, 1975, GA 24, p. 178. Heidegger finds in Kant the classic sentence in substantivization of the “I” pronoun, interpreted as self-consciousness: “The ‘I think' must be able to accompany all my representations” (*KrV*, § [13], B132).

^3^The ψ*υχ*ή is only the *o*ύσíα for those beings with life (ζωή). We prefer to remain dissatisfied and leave *o*ύσíα untranslated, since this automatically underscores the dissatisfaction with any of the classical translations.

^4^Aristotle, “Metaphysics,” 1017b16. In Π*ερ*í ψ*υχ*ής, Aristotle would go beyond the logical determination of the soul as ύπ*o*κεíμ*εν**o*ν of life and directly use the expression “principle” (άρχή): “for the soul is in some sense the principle of living beings” (De Anima, 402a6ff).

^5^Heidegger, 2002, GA 18, § [7], p. 33ff. Not in vain is the privileged place Aristotle reserves for his observations on self-consciousness (νóη*σις* ν*o*ήσ*εως*, as thinking of thinking) found within the framework of “Metaphysics” and not of “De Anima”; specifically, within the context of the last elucidation of eternal motion (“Metaphysics,” 1074b34). Analyses of the Π*ερ*í ψ*υχ*ής (“De Anima,” 429a10–430a10) are limited to framing comprehension of a general theory of sense knowledge.

^6^Descartes, *Oeuvres*, A&T, Vol. VII, Med. II, p. 27.

^7^Descartes, *Oeuvres*, A&T, Vol. VIII, par. LIII, p. 25. The Cartesian distinction between a single and plural meaning of the term “*substantia*,” according to which in a restricted use (substance is what does not need anything else to exist), could only apply to god; meanwhile, in a less strict use, it is applied to those substances that only depend on god to exist, that is those substances that have a relative and not absolute independence; see *Oeuvres*, A&T, Vol. VIII, LI, p. 24.

^8^Heidegger does not explicitly find this third step in Descartes but, rather, in Kant's expansion (Heidegger, GA 24, p. 177): The content of the *cogito* (*cogitationes*) in Kant becomes *determinationes* which are *praedicata* of things. The fact that the “I” must be able to accompany all my representations (now understood as *praedicata*) means that the “I” becomes “subiectum” in the formal apophantic sense, and this is the basis on which Kant's project is able to open up to a reframing of Aristotelian categories—a step that Descartes' ontology (embedded in the fundamental distinction between *res extensa* and *res cogitans*) did not need to even elaborate on.

^9^Heidegger, 1994, GA 17, § [46], p. 254.

^10^Heidegger, 1979, GA 20, § [11], p. 147.

^11^This has already been done successfully. For example, see Serrano De Haro (2006, pp. 103–114), Pereña (2008, pp. 39–54), and Benoist (1999, pp. 21–42).

^12^Zahavi, 2005, 2020.

^13^Heidegger, 1994, GA 17, § [48], p. 271.

^14^Husserl, 1991, pp. 100–103.

^15^With regard to *a priori* correlation, and from the many possible references, see Husserl, 1991, §§ [17], [27], [28]; Husserl, 1968, p. 290. Husserl is always much more emphatic when describing the impact of the discovery of *a priori* correlation on the confection of his philosophy than when describing the notion of intentionality inherited from F. Brentano; see, for example, Husserl, 1976b, § [48], p. 169: “The first breakthrough of this universal *a priori* of correlation between experienced object and manners of givenness affected me so deeply that my whole subsequent lifework has been dominated by the task of systematically elaborating on this *a priori* of correlation.” Intentionality gains meaning for Husserl (beyond its archaeology in Brentano's recovery of the notion based on ecclesiastical uses) in its strict fit as a vehicle for *a priori* correlation.

^16^In the parlance of Husserl's era, the “theory of wholes and parts.” Husserl obtains the concepts of “separability/inseparability” (*Abtrennbarkeit/Unabtrennbarkeit*) based on the analysis of independence/lack of independence (*Selbständigkeit/Unselbständigkeit*) of objects (see Husserl, 1984, III LU, § [3], p. 233).

^17^He defines it in contrast to alternative models of correlation, for example in Kant, which place it in the structure of judgement and its elements (“subject”—“object” correlation), and thus opens himself up to formal apophantic articulation and, with this, to the possibility of directly positing access to the problem of the self. In this sense, it would not be strange for Heidegger to progressively move away from intentionality (for him, from *SuZ*, fully absorbed in the practical approach to the world environment) and feel ever greater sympathy for Kant's proposition. For an evolution of Heidegger's interpretations of Kant, see H. Hoppe, in Klostermann (1970, pp. 284–317).

^18^Husserl locates an opposition to the transcendence of the world as a totality, the transcendence of god, an opposition that occurs “so to speak” (*gleichsam*) diametrically (Husserl, 1976a, § [58], p. 110). Husserl's insecurity in the metaphorical use of the expression of polarity likely places us in an initial, tentative, and imprecise use restricted to metaphorology, far from the operational uses that it would be destined to play in the theory of the pure ego.

^19^Husserl, 1952, § [25], p. 105. In “Ideas II,” the first appearance occurs in the title of §§ [22] and [23], yet not in the content (“The pure ego as ego-pole [Ichpol]” § [22]; “The possibility of grasping [*Erfassbarkeit*] the pure ego” § [23]). These appearances in the titles correspond to the inclusions Husserl added to the L. Landgrebe version in 1924/25. In general, Husserl introduces polarity in the discussion of the difficult problems of empathy and intersubjectivity; see Husserl, 1973b, Txt no. 2.

^20^In the volume, *Hua XXXVIII, Wahrnehmung und Aufmerksamkeit*, which contains Husserl's early lectures (up to 1912) dedicated to the phenomena of attention, the terminology of polarity barely appears, although the ray does, associated both to the ego (*Strahl des Ich*, p. 400) and intentionality (*intentionale Strahlen*) (Hua XXXVIII, *Beilage* XX, p. 316). He also postulates attention itself as a ray (Hua XXXVIII, *Beilage* XXI, p. 319), with the ego as the center from which these “rays of attention” emanate (p. 402), and this use (as a center of rays of attention) representing the clearest precursor of the “I-pole” notion.

^21^Husserl, 1973a, § [19], p. 155. The concept of the “pure ego” is introduced in *Grundproblemevorlesung* from 1910, within the context of the problem regarding the flow of consciousness of time, a problem which forced phenomenological reduction (performed on the immanent experience that is occurring) to become a “double reduction” (which is performed not only on the temporal flow of the immanent experience but also on the temporal flows of content reproduced in diverse presentifications, with their own courses of time: recollections, expectations, etc.). In this second reduction, I can address the backgrounds of those reproduced courses of time, for example (in one of the possible directions) the ego polarities on which those experiences are constituted, and verify how the simple possibility of intelligibility of that direction of attention represents a certain identity with regard to the ego polarity that is performed by reduction on immanent experience. In this sense, the “pure ego” in the area of double reduction is conceived as a possible result of the investigation into the problems of the consciousness of time.

^22^Husserl, 1973b, Txt no. 2, p. 30: “The I-pole is what it is, not a carrier, not a substratum for feeling and action, etc. but the ego as a point of radiance, as a function center of attention, as a point of emission; the center of activity for actions, for acts”; Husserl, 1973b, Txt no. 2, Appendix II, p. 43: “The ego is nothing more than the pole with no attribute of the acts, and it has all the determinations of this polarity, whereby the acts themselves are not something that is next to it, something comparable to this ego, with which, in a way, it is related” (author's translation).

^23^The fact that heterogeneity between the poles is irreducible closes the path to any attempt of identity between them, such as those attempts that aim to make this heterogeneity a mere moment of a dialectic process, where speculative idealism would be united, such as in Hegel.

^24^The ideation of the double directionality of intentionality is strengthened in the concept of polarization, which is less present where descriptions are only based on the phenomena of attention or in the different overlapping time frames in polythetic acts: a double direction is possible for each ray. In Husserl's terms, “two-fold radiations (*doppelte Strahlungen*), running ahead and running back: from the center outward, through the acts toward their Objects, and again returning rays, coming from the Objects back toward the center” (Husserl, 1952, § [25], p. 105). Thus, the intentional object is constituted as the “counter-pole” (*Gegenpol*) of the I-pole.

^25^Frequentative or iterative verbs are those that contain the notion of repetition or reiteration in their own semantics. They are formed in Latin through the participle of another verb, to which the suffix of repetition is added. The belligerent nuance of the tactile suggests the idea of an irregular or approximate reiteration, contained in the idea of “tentative”.

^26^The Latin verb “*apuntare*” indicates the frequentative idea of “prowling around a point,” whose active nature is contained in both the idea of the action itself and the Latin concept of *punctus*, the participle of *pungere*, meaning pierce or punch. The fixed “aim/direct” emerging from the idea of a frequentative suggests a genesis of fixation (of attention) always in a prior process of tentative “pecking”; we believe we found this idea in the marvelous work of Serrano de Haro on the phenomenology of aim, which he terms “informal calculation” (see, Serrano De Haro, 2007, p. 37ff).

^27^We are referring to the key point of typically monocular and physiological accommodation (seen in both eyes, taken independently). Accommodation is seen as a physiological key, as the information it provides refers to the oculomotor adjustments that control the position of the eyes: Ciliary muscle contraction leads to increased lens thickness, whereas relaxing the muscles reduces lens depth. Nonetheless, contraction and relaxation of the ciliary muscles depend on the size of the objects focussed on by the retina, whereby, through their combination, in particular with key binocular convergence (and others), it is responsible for depth perception as an “outcome”.

^28^By recovering the language of polarity for *intentio*, Husserl revitalizes, surely without intent, the original nature of its etymological meaning, even with regard to the original context where Franz Brentano recovered it: the framework of the problem of “intentional (or mental) inexistence of an object,” that is the problem of the “mode of existence” of immanent objects in consciousness. With regard to the subordination of categories of existence to the immanence–transcendence axis of the early scholastic presentations of intentionality, see Perler (2001, p. 203ff).

^29^The inclusion of this second sense for R. Ingarden represents a major advance with regard to presentations of the ego in “Ideas I”; see Husserl, 1991, p. 215.

^30^In the formal mereology of LU III, the “abstract” concept functions as a synonym for “non-independent part”; see Husserl, 1984, § [17], p. 273.

^31^Husserl, 1984, § [16], p. 271.

^32^Husserl, 1952, Appendix II to § [25], p. 310.

^33^This analogy appears in Husserl, 1952, § [25], pp. 106–107; Appendix II, p. 311; Husserl, 1973b, Txt no. 2, p. 30; Appendix IV, p. 50, and Husserl, 1973c, § [37], p. 131. The first text where this analogy is established is a manuscript based on Edith Stein's writing, in § [25] of “Ideas II”; for more on this manuscript, see Marbach (1974, p. 159). Husserl also widens the sense of the I as a substrate of habitualities to “infrahuman beings” (*untermenschlichen Personen*), a category that could include animals; for more on the phenomenology of animality in Husserl, see Javier San Martín (2007, p. 39). For a discussion on whether this analogy is more than a mere analogy, see Marbach (1974), § [25], p. 159ff and Micali, 2008, p. 27.

^34^The later text from 1931 is also important. Here, Husserl points to an identification between the words of “subject” and “center,” thus distancing the subject from the substrate functions of predication and reconciling it more with the sense of the pre-predicative I-pole: “‘Subject' is just another word to designate the centralization of all life as life of the ego” (author's translation; see, Husserl, 2006, Txt no. 10, p. 35).

^35^The concept of “ownership” is countered by that of “otherness” or “experience of the other.” For more on the relationship between the concept of “ownership,” which leads to primordial reduction, and intersubjective reduction, see González Guardiola (2021, p. 167ff).

^36^From such opposed extremes as “analytical Buddhism” (Albahari, 2006) or cognitive neuroscience (Metzinger, 2003).

^37^M. Ratcliffe, in Fuchs et al. (2017, p. 149ff).

^38^The best descriptions of the constitution process of the thing around an ego centralization are found in lessons from 1907 concerning “*Ding und Raum*”; see Husserl, 1973c, § [37], p. 131.

^39^Parnas and Sass (2001, p. 101ff).

^40^Husserl adds: “A question for further consideration (*näher zu überlegen*) would be how far one could progress along this path (*Wege*)”; see Husserl, 1952, § [25], p. 106. However, to which “path” of phenomenology does Husserl refer when being able to extend this exploration with a view to verifying its fecundity? It cannot be the Cartesian path and is unlikely to refer to the world of life or psychology. We will leave this consideration open at this time for subsequent research.

^41^We believe we have found the continuation of this “path” that leads to the phenomenological theory of orientation in some later texts by Hua XXXIX (texts 16, 20, and 21). As far as we know thus far, no attempt has been made to apply a completely systematic theory of orientation, with all the potential of the mereological tools in LU III, to the description of anomalous lived experiences for whose intelligibility the idea of the “minimal self” had been necessary.
